# The Effect of Flutamide on Ovulation Induction in PCOS Patients

**Published:** 2012-06-19

**Authors:** Tahereh Madani, Shohreh Irani Irani, Mahnaz Ashrafi, Maryam Alsadat Nabavi

**Affiliations:** 1 Department of Endocrinology and Female Infertility, Reproductive Biomedicine Research Center, Royan Institute for Reproductive Biomedicine, ACECR, Tehran, Iran; 2 Department of Reproductive Imaging, Reproductive Biomedicine Research Center, Royan Institute for Reproductive Biomedicine, ACECR, Tehran, Iran; 3 Epidemiology and Reproductive Health Department, Reproductive Biomedicine Research Center, Royan Institute for Reproductive Biomedicine, ACECR, Tehran, Iran

**Keywords:** Polycystic Ovary Syndrome, Flutamide, Ovulation Induction

## Abstract

**Background:**

Polycystic ovary syndrome (PCOS) is a disorder that affects various body organs and requires comprehensive long term evaluation and management. The aim of this study was to evaluate effect of Flutamide on ovulation induction in PCOS patients.

**Materials and Methods:**

This prospective study applied triple blind method, a simple convenience sampling method, to induce ovulations of the ninety six PCOS patients. Patients were divided into two groups; group A included 53 subjects (received Flutamide + Clomiphene Citrate) and group B included 43 subjects (received placebo + Clomiphene Citrate). Ultrasound was carried to determine the size of follicles and growth rate of them during follicular phase of the menstrual cycle. Also, progesterone levels were measured on days 19 and 21 of the menstrual cycle.

**Results:**

In this study, ninety six PCOS patients, in two treatment and control groups, were evaluated regarding to body mass index (BMI), cycle irregularity, age and number of dominant follicles, duration of stimulation, endocrine profile and score of hirsutism. The obtained results revealed no significant differences between two groups.

**Conclusion:**

Flutamide does not affect ovulation improvement in PCOS patients undergoing induction (Registration Number: IRCT 201105081141N10).

## Introduction

Polycystic ovary syndrome (PCOS) is a disorder that requires comprehensive long term evaluation and management which affects several body systems. It is observed in 5-7% of women in reproductive age and causes endocrinopathy in women (1).

It is a syndrome of hyperandrogenic chronic anovulation. Etiology of this syndrome is unknown and long term effects is poorly documented. Many symptoms characterize it and there is no certain clinical treatment, yet (2, 3). Diagnosis of poly cystic ovaries requires observation two of the three following criteria, by highlighting the range of clinical expressions within the disorder (as rotterdam criteria 2003) (2).

First, Poly cystic ovaries: when there are 12 or more follicles in each ovary, with diameter of 2-9 mm and/or ovarian volume >10 ml. Neither distribution of the follicles nor stromal density is included in this category. One polycystic ovary is sufficient for diagnosis. Second, oligo-/anovulation: it is clinically diagnosed as oligo-/amenorrhea which is menstrual cycles less than 10 times each year or longer duration than 35 days.

Third, Hyperandrogenism: in clinical or biochemical terms (2, 4), hirsutism is described as excess body hair in undesirable locations (5). The common method of assessing hirsutism is Ferriman-Gallway which is score describes four grades of hirsutism (5).

Approximately, %50 of women with PCOS are overweight or obese. Different studies have reported endocrine and metabolic differences between lean and obese PCOS women (1).

We investigated the effect of Flutamid and Clomiphen citrate in PCOS patients; Futamid is an oral nonsteroidal anti androgen drug along with clomiphen citrate stimulate the release of hormones needed for ovulation.

## Materials and Methods

In this prospective study, ninety six PCOS patients under age of 37 referred to Royan Research Center with history of infertility, irregular menstruation or hirsutism between 2005 to 2006. The subjects were studied by sampling method in treatment and control groups using Flutamide and Clomiphene Citrate or Placebo and Clomiphene Citrate, respectively. The study was approved by Institutional Review Board of Royan Institute Research Center and Royan Ethics Committee. The patients signed consent forms before enrolling in the research and treating with Clomiphene Citrate. Discontent of attending, being allergic to the drugs (Flutamide or Clomiphene Citrate), hyperprolactinemia, thyroid dysfunction, age over 37, and body mass index (BMI) > 30 were the causes to exclude of the study. Estradiol, testosterone, prolactin, thyroid tests, hepatic tests, and luteinizing hormone (LH)-follicle stimulating hormone (FSH) were taken from all patients on day three of their menstruations followed by signing the consent forms. Placebo samples (Daroupakhsh Pharmaceutical Company, Iran) were prepared and confidentially coded by an individual from out of the research team. Then, the patients were randomly divided into two groups, A (53 subjects) and B (43 subjects). Triple-blind method was applied for the treatment group. Group A daily received Flutamide tab 250 mg (Pharmascience, Canada), started on day 3 till end of their menstrual cycles. Group B received placebo, started on day 3 till end of their menstrual cycles. This continued for their next 2 cycles. Also, Clomiphene Citrate 50 mg (Parsminoo- Iran) was given to both groups, started on day 3 to day 7 of their menstrual cycles and it was carried on for two consecutive cycles. The dosage of Clomiphene Citrate was gradually increased, the patients daily received Clomiphene Citrate 100 mg from day 3 to day 7 in the first cycle; while, the daily dose from day 3 to day 7 changed to 150 mg for the second cycle. However, group A and B used fixed dose of Flutamide and Placebo.All the patients were monitored by a sonography.

When follicle size reached ≥18 mm, human chorionic gonadotrophine (hCG) was injected up to 10/000 units followed by measuring progesterone on days 19 and 21 of their menstruations to determine ovulation. These two therapeutic cycles were continued with patient agreement. Ovulation in two groups was studied at the end of the second cycles. Furthermore, considerable effects of Flutamide on ovulation were measured through different statistical tests between both groups. Hirsutism was also assessed in both group using Ferriman-Gallway score (4). In this study, we divided the patients into grade 1 with low hirsutism or grade 2 with high hirsutism depended on Ferriman-Gallway scores from four different regions: upper lip, chin, lower abdomen, and thighs. Also, patients with BMI rate ≤ 25 were considered normal and over 25 were considered obese. The analysis was done by SPSS version 16.0, Mann-Whitney test and chi-square test.

## Results

Ninety and six PCOS patients, age of 18 - 37, were participated in this research. Mean age was 26.96 years old. The mean of age, BMI, duration of infertility, LH, basic estradiol, thyroid stimulating hormone (TSH), T4, T3, prolactin, testosterone, progesterone on days 19 and 21 of their menstruations, size of follicles and volume of ovaries are separately shown in the table 1. It is important to consider that total patients participated in the first cycle were 96. Since some of the patients were not willing to continue treatment, so there were only 69 patients in the second cycle.

According to the data shown on tables 2, 3 and 4, Flutamide had no effect on induction of ovulation based on BMI and hirsutism in both groups in these two therapeutic cycles. Flutamide had no effect on regulation of menstruation (ovulation), mean size of follicle, number of dominant follicle, and the length of stimulation period in two therapeutic cycles. By taking ovulation induction into account, hirsutism had no meaningful difference in two therapeutic cycles in A and B groups.

**Table 1:** Characteristics of patients


Variable	Group A Mean ± SE	Group B Mean ± SE	P value

**Age (Years)**	27.19± 0.53	26.65± 0.65	NS
**BMI (Kg/m^2^)**	27.51± 4.29	27.27± 4.26	NS
**Infertility duration (Years)**	6.5 ± 0.5	5.78± 0.43	NS
**Menstruation**			
Regular	17 (32.1%)	15 (34.9%)	NS
Irregular	36 (67.9%)	28 (65.1%)	NS
**Level of LH (mIU/ml)**	7.78± 0.78	7.88± 0.9	NS
**Level of basal estradiol (pg/ml)**	77.9± 10.2	102.87 ± 24.1	NS
**T3 (ng/ml)**	19.75± 5.9	18.53± 6.98	NS
**T4 (ng/dl)**	20.35± 4.99	22.85± 5.44	NS
**TSH (mIU/l)**	1.97± 0.19	1.79± 0.22	NS
**Prolactin (mIU/l)**	196.9± 34.1	189.1± 57.97	NS
**Testosterone (ng/ml)**	2.79± 2.1	2.95± 2.1	NS
**Progesterone level on day 19 (ng/ml)**	1.9 ± 0.61	2.85± 0.86	NS
**Progesterone level on day 21 (ng/ml)**	2.69± 0.86	3.55± 1.17	NS
**Volume of right ovary (cm^3^)**	9 ± 0.53	8.4 ± 0.47	NS
**Volume of left ovary (cm^3^)**	8.1 ± 0.54	7.66± 0.52	NS


NS; Non significant.

**Table 2:** Outcome cycle 1, 2


	Group A Mean ± standard error (SE)		Group B Mean ± standard error (SE)	P value

**Duration of stimulation**				
**Cycle 1**	15.45± 0.54		14.83 ± 0.36	NS
**Cycle 2**	14.6 ± 0.62		14.44 ± 0.49	NS
**Size of dominant follicles**				
**Cycle 1**	18.96± 0.63		19.33 ± 0.57	NS
**Cycle 2**	19.41± 0.7		18.37 ± 0.57	NS


NS; Non significant.

**Table 3:** Outcome with regard to physical characteristics cycle 1


	Group A Ovulation		Group B Ovulation		P value
	Yes	No	Yes	No		

**Patients**	33(63.5%)	19(36.5%)	26 (60.5%)	17 (39.5%)	
**BMI (Kg/m^2^)**							
**Normal≤25**	12(80.0%)	3 (20.0%)	10 (55.6%)	8 (44.4%)	NS
**Obese >25**	21(56.8%)	16(43.2%)	16 (64.0%)	9 (36.0%)	NS
**Hirsutism**							
**Grade 1**	13	(52%)	12(48%)	11(57.9%)	8 (42.1%)	
**Grade 2**	19	(73.1%)	7 (26.9%)	13 (59.1%)	9 (40.9%)	NS


NS; Non significant.

**Table 4:** Outcome with regard to physical characteristics cycle 2


Variables	Group A Ovulation		Group B Ovulation		P value
	Yes	No	Yes	No	

**BMI (Kg/m^2^)**						
**Normal≤25**	10(55.6%)	8 (44.4%)	12 (70.6%)	5 (29.4%)	NS
**Obese >25**	9 (42.9%)	12(57.1%)	7 (53.8%)	6 (46.2%)	NS
**Total**	19(48.7%)	20(51.3%)	19 (63.3%)	11 (36.7%)	NS
**Hirsutism**						
**Grade 1**	12(57.1%)	9 (42.9%)	8 (66.7%)	4 (39.3%)	NS
**Grade 2**	6 (35.3%)	11 (64.7%)	11 (61.1%)	7 (38.9%)	


NS; Non significant.

## Discussion

In obese and normal weight patients with PCOS, Flutamide has been studied as an anti-androgenic drug to improve ovulation. The results of some studies have shown that low dose of Flutamide/ Metformin/ Oral Contraceptive Pill (OCP) in young PCOS patients with normal weight improves the resistance to insulin and dyslipidemia which are long-term side effects of PCOS administration. Therefore, this compound is an appropriate treatment for young and thin PCOS women (6). Flutamid reduces adrenal androgen production in obese women with PCOS, but has no effect on lipid spectrum or insulin resistance (7).

Effective role of Clomiphene Citrate has been long known. Thus, our intentional is to study the effect of Flutamide along with clomiphene on ovulation induction.

Despite other studies indicate Flutamide (6-13) plays an effective role on ovulation induction, the present study shows that Flutamide is not effective on ovulation induction in A and B groups in two therapeutic cycles. In the mentioned studies, OCP and Metformin were prescribed to the treatment group for longer time period which it may explain ineffectiveness of Flutamide in our study.

In this study, Flutamide is not effective in regulating cycles and improvement of ovulation in two therapeutic cycles in A and B groups. However, Flutamide is effective element in other studies (6, 11) which may be due to taking Metformin and OCP along with Flutamide. In other studies (9-13), Flutamide plays an effective role on reduction of Testosterone following by diminishing hirsutism, but this is not in agreement with our results. Ovulation induction, hirsutism were the same in both groups and no significant difference is shown. Mean size and number of dominant follicles in two therapeutic cycles between the two groups and period of stimulation can be referred as other findings of this study and no significant difference is shown.

## Conclusion

Our study showed that Flutamide has no effect on improving ovulation in patients with the history of PCOS undergoing induction.
